# Survival rates of patients who undergo minimally invasive surgery for endometrial cancer with cervical involvement

**DOI:** 10.7150/ijms.55026

**Published:** 2021-03-21

**Authors:** Sang Il Kim, Dong Choon Park, Sung Jong Lee, Min Jong Song, Chan Joo Kim, Hae Nam Lee, Joo Hee Yoon

**Affiliations:** 1 Department of Obstetrics and Gynecology, St. Vincent's hospital, College of Medicine, The Catholic University of Korea, Seoul, Republic of Korea.; 2 Department of Obstetrics and Gynecology, Seoul St. Mary's hospital, College of Medicine, The Catholic University of Korea, Seoul, Republic of Korea.; 3 Department of Obstetrics and Gynecology, Yeouido St. Mary's hospital, College of Medicine, The Catholic University of Korea, Seoul, Republic of Korea.; 4 Department of Obstetrics and Gynecology, Uijeongbu St. Mary's hospital, College of Medicine, The Catholic University of Korea, Seoul, Republic of Korea.; 5 Department of Obstetrics and Gynecology, Buchen St. Mary's hospital, College of Medicine, The Catholic University of Korea, Seoul, Republic of Korea.

**Keywords:** endometrial cancer, cervical involvement, minimally invasive surgery, laparoscopy, LACC trial

## Abstract

**Objective:** Compare the oncologic outcomes of patients with intermediate-risk endometrial cancer who were staged by minimally invasive surgery with the outcomes of patients who underwent open surgery.

**Methods:** Data from 206 patients with intermediate-risk endometrial cancer who were treated between January 2009 and January 2019 were reviewed. The patients' data were retrieved from five institutions. The patients were divided into two groups: those who underwent open surgery and those who underwent minimally invasive surgery. Tumor characteristics, recurrence rate, disease-free survival, and overall survival were compared according to surgical approach.

**Results:** Among the 206 patients included in this study, 76 underwent open surgery (36.9%) and 130 underwent MIS (63.1%). In patients with stage IB endometrial cancer, the recurrence rate, disease-free survival, and overall survival were not significantly different between those who underwent minimally invasive surgery and those who underwent open surgery. However, in patients with stage II endometrial cancer, the recurrence rate was significantly higher among those who underwent minimally invasive surgery (37.5% vs. 5.3%, p = 0.013). Patients with stage II endometrial cancer who underwent minimally invasive surgery had a significantly lower disease-free survival (p = 0.012) than those who underwent open surgery, however, the overall survival (p = 0.252) was similar between the two groups.

**Conclusion:** Minimally invasive surgery results in less favorable survival outcomes than open surgery in patients with stage II endometrial cancer.

## Introduction

Endometrial cancer (EC) is the most common gynecologic cancer in developed countries 1. In the United States, approximately 65,620 new cases of EC and 12,590 have been projected in 2020 2. In Korea, the incidence of EC has been increasing, with a projection of 3261 new cases and 383 deaths, and it is the most common gynecologic cancer 3, 4.

Surgical staging, including total hysterectomy and bilateral salpingo-oophorectomy with lymph node assessment is recommended for patients with EC 5, 6. Since the adoption of minimally invasive surgery (MIS) for EC, the incidence of MIS compared to open surgery has gradually increased 7. MIS results in oncologic outcomes similar to those by open surgery, with reduced surgical morbidity and a better quality of life 8-16. Thus, MIS is accepted as a treatment option for EC, and current guidelines from the National Comprehensive Cancer Network (NCCN) indicate that both open surgery and MIS are acceptable approaches for uterine cancer 6. However, in November 2018, results from the Laparoscopic Approach to Cervical Cancer (LACC) trial indicated that patients undergoing MIS have a lower rate of disease-free survival and overall survival than those who undergo open surgery 17. Although randomized clinical trials have shown that survival after MIS is similar to survival after open surgery among patients with early stage EC, the safety of this approach has been controversial since the unexpected results of the LACC trial, especially in patients with EC with cervical involvement.

According to the International Federation of Gynecology and Obstetrics (FIGO) staging system, EC with cervical involvement is defined as stage II 18. The Gynecologic Oncology Group (GOG)-99 study defined stage IB, IC, and II EC as the intermediate-risk group 19. However, the GOG criteria followed the 1998 FIGO staging system, and are therefore difficult to use. Thus, *Kong et al.* reported a validation study of the GOG criteria and proposed simplified criteria 20. In this study, stage IB and II EC were defined as the intermediate-risk group.

The aim of this retrospective, multicenter study was to compare the oncologic outcomes of patients with intermediate-risk EC who were staged by MIS with the outcomes of patients who underwent open surgery.

## Materials and Methods

This retrospective, multicenter study was performed with approval from the institutional review board of The Catholic University of Korea (No. XC20RADI0115V). The requirement of informed consent was waived due to the nature of the study. The study was conducted in accordance with the Declaration of Helsinki.

Data from 206 patients with intermediate-risk EC who were treated between January 2009 and January 2019 were reviewed. The patients' data were retrieved from five institutions: Seoul St. Mary's Hospital (n=98), St. Vincent's Hospital (n=40), Yeouido St. Mary's Hospital (n=17), Uijeongbu St. Mary's Hospital (n=28), and Bucheon St. Mary's Hospital (n=23). We reviewed the patients' medical records, pathologic reports, imaging studies, and clinicopathologic characteristics (age, BMI, histologic type, grade, FIGO stage, tumor size, and risk factors identified by pathological examination). Only patients who underwent primary surgery were eligible. Both pelvic and para-aortic lymphadenectomy were recommended. However, when pelvic lymph nodes were free of the disease, para-aortic lymphadenectomy could be omitted. The patients were divided into two groups: those who underwent open surgery and those who underwent MIS. Robot-assisted surgery was included in the MIS group. After surgery, adjuvant radiotherapy was selectively administered, according to the stage and presence of risk factors (age, grade, lymphovascular space invasion, and tumor size). The uterine manipulator was used on a case-by-case basis. In addition, patients diagnosed with EC commonly underwent preoperative magnetic resonance imaging (MRI) and/or positron emission tomography-computed tomography (PET-CT) to assess the tumor's local invasion, lymph node metastasis, and distant spread.

Overall survival (OS) was calculated from the date of the initial diagnosis to the cancer-related death or the last follow-up. Disease-free survival (DFS) was calculated from the date of the initial diagnosis to the date of first disease progression or death.

The clinocopathological characteristics of the two groups were also compared. We used the Student's t-test, chi-square test, or Fisher's exact test to compare variables. The Kaplan-Meier method was used to compare survival outcomes between the two groups. All statistical analyses were performed using SPSS statistical software (version 21.0; SPSS Inc., Chicago, IL, USA). Statistical significance was set at P < 0.05.

## Results

Among the 206 patients included in this study, 76 underwent open surgery (36.9%) and 130 underwent MIS (63.1%). In the MIS group, 111 patients (85.4%) were scheduled for traditional laparoscopy and 19 (14.6%) were scheduled for robotic surgery. The rate of MIS showed no significant differences between five institutions: Seoul St. Mary's Hospital (60.2%), St. Vincent's Hospital (67.5%), Yeouido St. Mary's Hospital (58.8%), Uijeongbu St. Mary's Hospital (n=60.7%), and Bucheon St. Mary's Hospital (65.2%). The clinicopathologic characteristics of the patients are presented in Table 1. The mean age of patients in the MIS group was 59 years, and the mean body mass index (BMI) was 25.1 kg/m^2^. The mean age of the open surgery group was 58 years, and the mean BMI was 25.9 kg/m^2^. In the open surgery group, 57 patients (75.0%) were classified with stage IB EC, and in the MIS group, 106 patients (81.5%) were classified with stage IB EC. No significant differences were observed between the two groups in histologic subtype and grade. In entire cohort, about 60% of patients received both pelvic and para-aortic lymphadenectomy, and the two groups were comparable in terms of lymphadenectomy status. Patients who underwent open surgery had significantly larger tumors (median size 4.8 vs. 3.9 cm, p = 0.008) and positive lymphovascular space invasion (LVSI) (39.5% vs. 22.3%, p = 0.011). The open surgery group had a higher rate of patients receiving adjuvant therapy (84.2% vs. 66.2%, P = 0.006).

**Figure 1:**
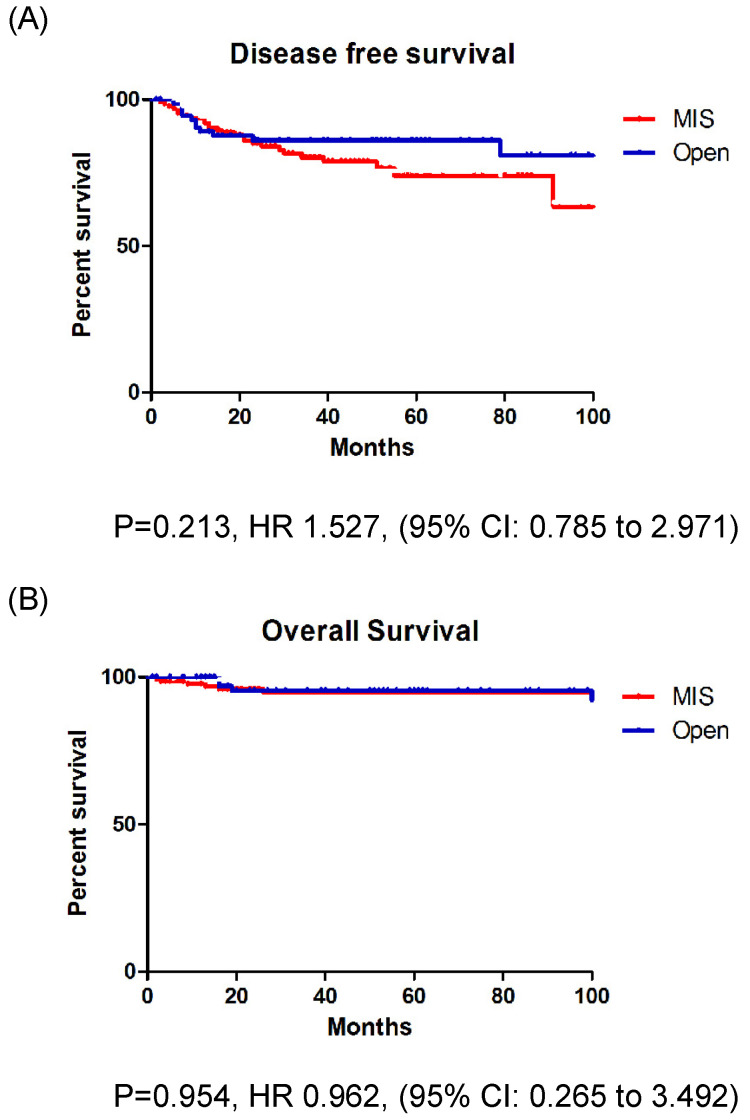
A: Disease free survival in entire cohort. B: Overall survival in entire cohort.

During a median observation period of 48 months (range: 1-129 months), 37 patients (18.0%) experienced disease recurrence (Table 2). Recurrences occurred in 26 patients (20.0%) who underwent MIS and 11 patients (14.5%) who underwent open surgery. The recurrence rate was higher in the MIS group, but there was no significant difference between the groups (p = 0.318). Relapse location was not affected by the surgical approach (p = 0.980). There were 11 cancer-related deaths (5.3%): 6 (4.6%) in the MIS group and 5 (6.6%) in the open surgery group (p = 0.538). The DFS (p = 0.213) and OS (p = 0.954) rates were similar between the two groups (Fig. 1A, B).

**Figure 2:**
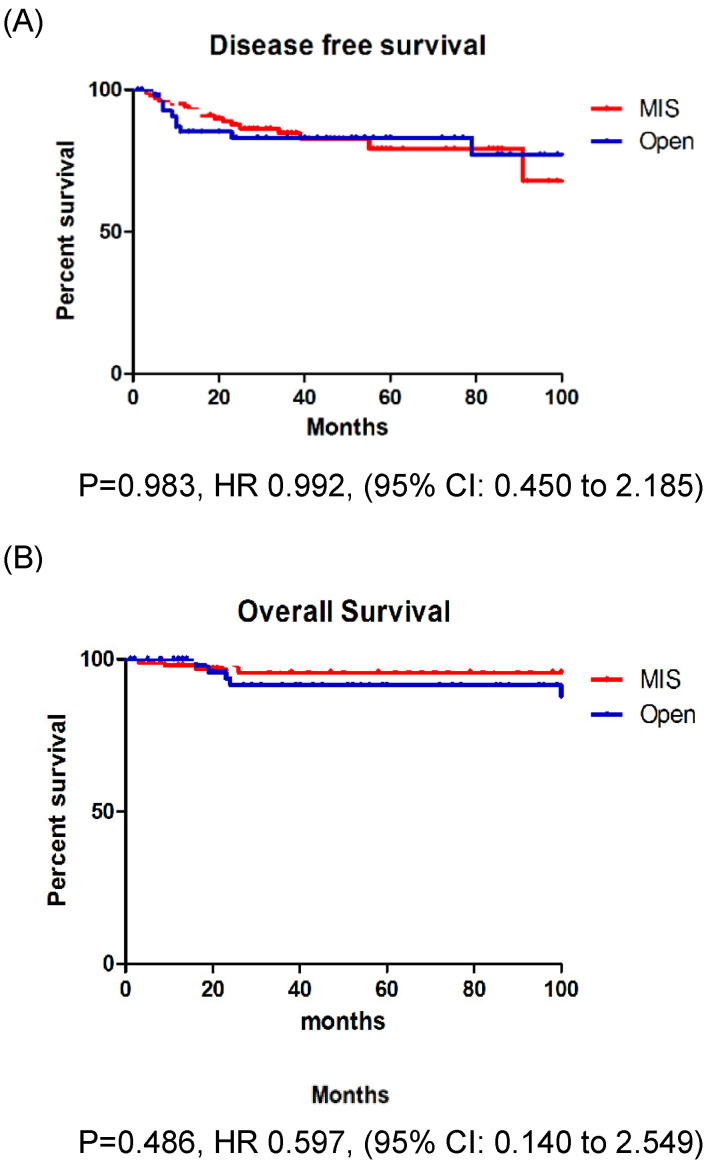
A: Disease free survival in stage IB patients. B: Overall survival in stage IB patients.

**Figure 3:**
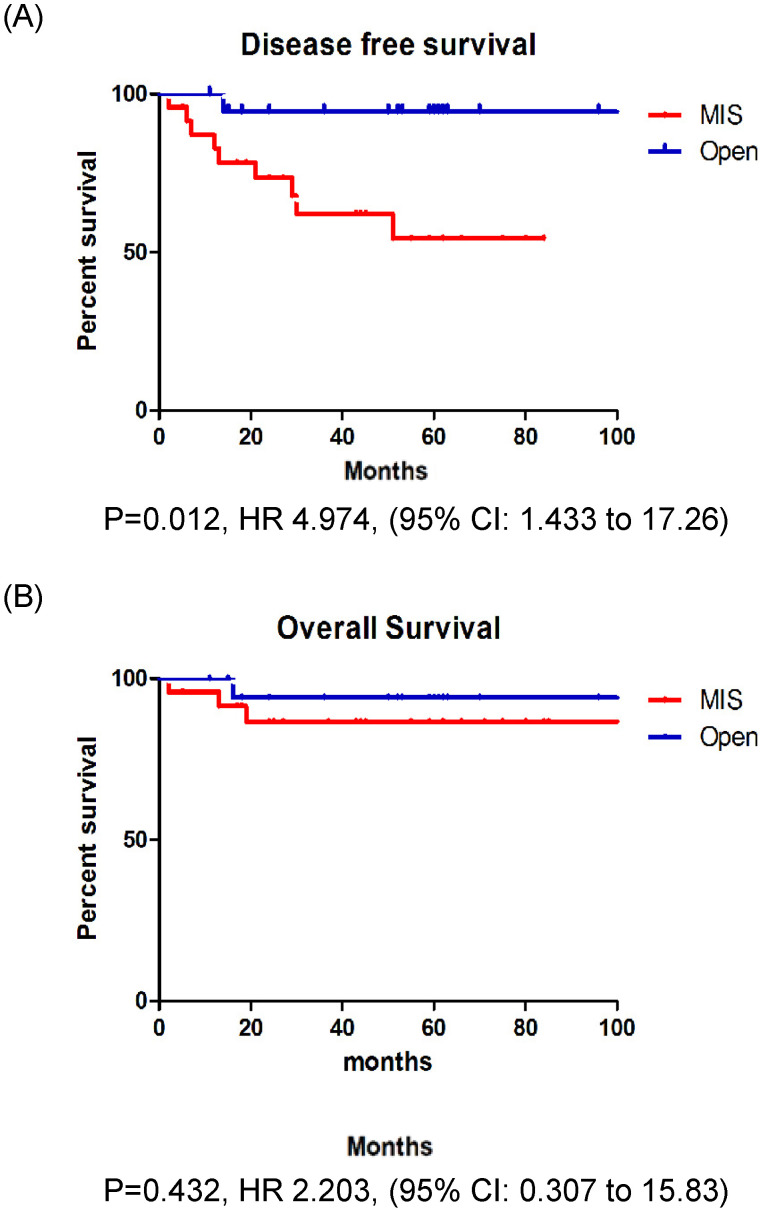
A: Disease free survival in stage II patients. B: Overall survival in stage II patients.

In patients with stage IB EC, the recurrence rate, DFS, and OS were not significantly different between those who underwent MIS and those who underwent open surgery (Fig. 2A, B). However, in patients with stage II EC, the recurrence rate was significantly higher among those who underwent MIS (37.5% vs. 5.3%, p = 0.013). Patients with stage II EC who underwent MIS had a significantly lower DFS (p = 0.012) than those who underwent open surgery, however, the OS (p = 0.252) was similar between the two groups (Fig. 3A, B).

## Discussion

This study evaluated the oncologic safety of MIS in patients with intermediate-risk EC. We found that patients who underwent MIS experienced similar recurrence and survival outcomes as patients who underwent open surgery. However, among patients with stage II EC, a higher recurrence rate and poorer DFS outcomes occurred in patients who underwent MIS.

The first laparoscopic surgery for EC was reported in 1992 21. Since then, many studies have reported the efficacy of MIS for EC, including randomized trials and a systemic review of the Cochrane Database 8-16, 22. These studies found that MIS is associated with lower complication rates and decreased hospital stays, without compromising oncologic outcomes.

Two large randomized trials comparing the outcomes of MIS and open surgery for EC have been conducted: the LAP2 study (n = 2,616) and the LACE trial (n = 760) 14, 16. The LAP2 study included patients with clinical stage I to IIA disease, which is classified as stage I in the revised FIGO staging system. The recurrence and overall survival rates were not different between patients who underwent MIS and patients who underwent open surgery. The LACE trial also included patients with stage I disease, and found no significant differences in recurrence or overall survival between the two groups. Both of these trials focused on patients with low-risk EC.

However, MIS was found to have inferior outcomes compared to open surgery in a recent, randomized trial of patients with cervical cancer 17. These results led to controversy on the benefits of MIC in gynecologic oncology patients, as it was believed that similar outcomes would occur in patients with EC with cervical involvement. Thus, we compared the outcomes of MIS and open surgery for the treatment of stage II EC. We included stage IB EC, as stage IB and stage II are both regarded as intermediate-risk EC 19, 20.

Several retrospective studies have shown that a high-risk histologic subtype is not a contraindication to minimally invasive surgery 23-25. Therefore, we included all histologic subtypes in our study. We found that histologic subtype does not affect the recurrence or survival of patients undergoing MIS or open surgery for EC.

In this study, open surgery group had significantly larger tumors and positive LVSI, and this could be related to the higher rate of adjuvant radiotherapy in the open surgery group.

Patients with stage II EC who underwent MIS had a higher recurrence rate and poorer DFS in this study. These differences were not observed among patients with stage IB EC in this study. The major difference between these two subtypes is cervical involvement. And following the NCCN guidelines, patients with stage II EC received radical hysterectomy 6. The difference in recurrence rates and DFS may be explained by several factors. First, an intracorporeal colpotomy can lead to tumor exposure and promote dissemination. Second, a stiff trendelenburg down position during MIS can also cause tumor spillage into the pelvic and abdominal cavity. These surgery- or surgeon-related factors may cause inferior oncologic outcomes in the MIS group. Thus, it is important to focus on the prevention of possible tumor spillage during MIS.

*Kanao et al.* reported a no-look no-touch technique to prevent intraoperative tumor spillage in early stage cervical cancer. They compared the surgical and oncologic outcomes of total laparoscopic radical hysterectomy with the no-look no-touch technique to abdominal radical hysterectomy; oncologic outcomes were similar in both groups 26. We think this technique also can be used in patients with EC to prevent tumor spillage, especially when cervical involvement is suspected.

Before making a decision on the surgical approach, the evaluation of cervical involvement is essential. MRI is the most reliable imaging technique for the diagnosis, staging, treatment planning, and follow-up of EC. The diagnostic accuracy of MRI in the assessment of cervical invasion has been reported to be approximately 90% 27, 28.

Our study is not without limitations. First, due to the retrospective study design, inevitable issues such as selection bias may exist. Second, the sample size may be insufficient to properly compare DFS and OS between the two groups. Third, variations in technique, expertise, and outcomes among surgeons at the five participating institutions were not considered. Fourth, the operative morbidity was not evaluated for either surgical approach.

In conclusion, MIS results in less favorable survival outcomes than open surgery in patients with stage II EC. The preoperative evaluation of cervical involvement is necessary when planning the surgical approach. MIS should be performed in carefully selected patients with precise surgical techniques.

## Figures and Tables

**Table 1:** Clinopathological characteristics of patients

	Open(n = 76, %)	MIS(n = 130, %)	Total(n = 206, %)	P value
Age, mean	57.9	59.4	58.8	0.321
BMI (kg/m^2^), mean	25.9	25.1	25.4	0.150
**FIGO stage**				0.267
IB	57 (75.0)	106 (81.5)	163 (79.1)	
II	19 (25.0)	24 (18.5)	43 (20.9)	
**Grade**				0.564
1	25 (32.9)	30 (23.1)	55 (26.7)	
2	29 (38.1)	60 (46.1)	89 (43.2)	
3	10 (13.2)	24 (18.5)	34 (16.5)	
Other high grade*	12 (15.8)	16 (12.3)	28 (13.6)	
Tumor size (cm), median	4.8	3.9	4.4	0.008
Range	1.5-10.2	0.8-9.3	0.8-10.2	
LVSI positive	30 (39.5)	29 (22.3)	59 (28.6)	0.011
**Lymphadenectomy**				0.417
Pelvic only	33 (43.4)	49 (37.7)	82 (39.8)	
Pelvic and para-aortic	43 (56.6)	81 (62.3)	124 (60.2)	
**Adjuvant treatment**				0.006
Radiotherapy	64 (84.2)	86 (66.2)	150 (72.8)	
None	12 (15.8)	44 (33.8)	56 (27.2)	

BMI, body mass index; FIGO, International Federation of Gynecology and Obstetrics; LVSI, lymphovascular space invasion; *Serous, clear cell, carcinosarcoma, dedifferentiated, undifferentiated.

**Table 2:** Oncologic outcomes of patients

	Open (n = 76, %)	MIS (n = 130, %)	Total (n = 206, %)	P value
Recurrences, total	11 (14.5)	26 (20.0)	37 (18.0)	0.318
**Recurrences by stage**				
Stage IB	10/57 (17.5)	17/106 (16.0)	27/163 (16.6)	0.805
Stage II	1/19 (5.3)	9/24 (37.5)	10/43 (23.3)	0.013
**Recurrences by grade**				
G1	1/25 (4.0)	6/30 (20.0)	7/55 (12.7)	0.112
G2	2/29 (6.9)	11/60 (18.3)	13/89 (14.6)	0.208
G3	3/10 (30.0)	6/24 (25.0)	9/34 (26.5)	0.763
Other high grade	5/12 (41.7)	3/16 (18.8)	8/28 (28.6)	0.231
**Recurrence site**				0.980
Stump	2	3	5	
Peritoneum	1	4	5	
Lymph node	1	5	6	
Distant metastasis (lung, bone, brain)	7	13	20	
Median follow-up (months)	54.9	43.9	48.0	0.017
Range	1-129	1-127	1-129	
Death	5 (6.6)	6 (4.6)	11 (5.3)	0.538

BMI, body mass index; FIGO, International Federation of Gynecology and Obstetrics; LVSI, lymphovascular space invasion; *Serous, clear cell, carcinosarcoma, dedifferentiated, undifferentiated.
